# Residential segregation, neighborhood violence and disorder, and inequalities in anxiety among Jewish and Palestinian-Arab perinatal women in Israel

**DOI:** 10.1186/s12939-020-01339-5

**Published:** 2020-12-09

**Authors:** Nihaya Daoud, Samira Alfayumi-Zeadna, Aviad Tur-Sinai, Nabil Geraisy, Ilan Talmud

**Affiliations:** 1 Department of Public Health, Faculty of Health Sciences, Ben-Gurion University of the Negev, P.O. Box 653, 84015 Beer Sheva, Israel; 2 Department of Health Systems Management, The Max Stern Yezreel Valley College, Yezreel Valley, Israel; 3 Department of Psychiatry, EMMS Nazareth Hospital, Nazareth, Israel; 4 Department of Sociology, University of Haifa, Haifa, Israel

**Keywords:** Residential segregation, Neighborhood violence and disorder, Anxiety, Inequalities, Women, Perinatal, Mental health, Discrimination, Collective efficacy, Stress, Socioeconomic status

## Abstract

**Background:**

Residential segregation can foster health inequality mechanisms by increasing stress related to neighborhood violence and disorder.

**Aims:**

We studied the association between neighborhood violence and disorder and inequalities in anxiety between two groups of perinatal Israeli women (Jewish, Palestinian-Arab) living in ethno-nationally segregated neighborhoods, and explored the influence of neighborhood characteristics; social support and chronic stress to this inequality.

**Methods:**

We linked survey data on neighborhood violence and disorder, neighborhood social characteristics (collective efficacy, social capital and social support) and aggregate discrimination to neighborhood SES census data. The survey data was obtained from the “Family Relations, Violence and Health” study (2014–2015) and included a stratified national sample of women (Palestinian-Arab = 436, Jewish = 965) residing in 63 segregated neighborhoods. We conducted multi-variable logistic regression analysis for anxiety (measured based on State-trait Anxiety Inventory) using generalized estimating equation (GEE) to estimate odds ratios of the association with neighborhood violence and disorder (total score for 10 problems) while considering neighborhood characteristics (SES; social characteristics; aggregate discrimination), social support and chronic stress in different models for the total sample, and separately for Palestinian-Arab and Jewish women.

**Results:**

Palestinian-Arab women had higher anxiety (60.5% vs. 42.1%, respectively) and higher severity of neighborhood violence and disorder (49.5% vs. 16.2%, respectively) compared to Jewish women. After considering individual and neighborhood variables, adjusted odds ratio (AOR) and 95% confidence intervals (CI) = 1.63, 1.04–2.56. The association between neighborhood violence and disorder and anxiety was significant for low vs. no problems in the final model for the total sample (AOR, 95%CI = 1.28, 1.00–1.64). Similarly, significant association was found only for low severity vs. no problems for Jewish women (1.40, 1.07–1.86). While among Palestinian-Arab women the association between neighborhood violence and disorder and anxiety rendered insignificant in the final model. Neighborhood social cohesion and social support were protective factors from anxiety in both groups, high neighborhood SES was protective factor only among Jewish women, and neighborhood aggregate discrimination was a risk factor only in Palestinian-Arab women.

**Conclusions:**

Inequalities in anxiety related to neighborhood violence and disorder in ethno-national perinatal groups of women likely reflect residential segregation. Policies entrenching segregation might have affected neighborhood mechanisms (SES inequalities, aggregate discrimination and low social cohesion) that lead to higher stress and ethno-national inequalities in anxiety among perinatal women.

## Introduction

Residential segregation generally refers to the physical separation of two or more ethnic groups into different neighborhoods within a specified geographic area, such as a municipality or metropolitan area [3, 49]. Residential segregation denotes the extent to which groups of different racial, ethnic, or national origins live in separate neighborhoods. However, groups can also be residentially segregated on the basis of religion, socioeconomic status (SES), education, unemployment, social assistance, country of birth and immigrant background [52]. Residential segregation plays an important role in social exclusion. Thus, low and moderate levels of residential segregation, as opposed to living in a multi-ethnic neighborhoods can increase social exclusion of ethnic minority groups and reduce their contact with other groups [43]. As a result, residential segregation plays a unique role in the formation of disadvantage and, by extension, clustering of poor health among residents. Residents confined to disadvantaged neighborhoods are uniquely at risk of health disparities, high poverty, loss of social stability and cohesion [28].

One mechanism through which neighborhood segregation affects health and health inequalities is neighborhood violence and disorder [24, 44]. Violence is a global public health problem affecting millions of people and resulting in half a million deaths every year [8]. Previous research showed that neighborhood violence was associated with poor health [24], including depression and anxiety [13, 32], and might contribute to inequalities in health [24]. Yet, despite interest in the effects of the living environment on mental health dating back to ecological theories of public health of the nineteenth century [48], the effects of residential segregation via neighborhood violence and disorder on inequalities in health are still not clear.

While neighborhood violence and disorder might affect all residents, perinatal women might be particularly susceptible. Neighborhood violence and disorder might intensify stress and amplify existing levels of higher anxiety related to women’s multiple roles during pregnancy, childbirth and early child rearing [22]. However, few studies have examined the relationships between neighborhood violence and disorder and anxiety in perinatal women, with even fewer comparing this relationship in minority women living in segregated neighborhoods [33, 51]. The current study examines inequalities in anxiety related to neighborhood violence and disorder between two ethno-national groups of women living in segregated neighborhoods and explores neighborhood-level factors contributing to this health inequality.

### Neighborhood violence and disorder and inequalities in anxiety

Anxiety, a common mental health disorder [54], is highly prevalent among perinatal women [22]. Although anxiety disorders have specific parameters, common symptoms include excessive and intrusive worrying, feeling overwhelmed, angry or scared, irritability, fatigue, difficulty concentrating and sleeping, elevated sensitivity to threat, and a bias towards negatively interpreting ambiguous information [4].

Among women, prevalence of anxiety symptoms appears to be high during the perinatal period (5 to 19%), depending on the week of pregnancy or postnatal period [22]. Some research suggests that neighborhood violence and disorder is associated with mental health problems and anxiety in women. Researchers in the UK used longitudinal data to show that local crime was associated with increased depression and anxiety among women [25]. A study in the US that included a sample of 386 women showed that those who witnessed community-level violence were twice as likely to have depression and anxiety without direct contact with violence [12]. Another two studies in the US found associations between neighborhood violence and women’s health, including depression [33, 51]: the first drew on a sample of unmarried mothers and looked at parenting [33], while the other used a small sample of low-income mothers [51]. However, we know of few studies examining the impact of neighborhood disorder on inequalities in anxiety in women.

Previous research showed higher rates of anxiety in minority women compared to other groups [23]. For example, anxiety was high among Asian-American women in the US [42], and immigrant women in the UK [55] and Canada [23]. Further, research into individual-level factors showed that low social support and discrimination [6], SES and substances use [70], as well as adaptation to a host society involving new language use, social norms, and living settings (rural to urban) [26] were associated with increased anxiety for minority women. Thus, multiple individual-level risk factors might combine to increase anxiety among minority women, in particular, during the perinatal moment. But little is known about the contribution of neighborhood-level factors to this inequality. Whitley and Prince [72] did find that low-income women who reported higher neighborhood crime experienced more barriers to accessing and using their local and wider environments, which might increase stress and adversely affect mental health [72]. Meanwhile, studies of neighborhood effects have shown that neighborhoods characterized by low income and high crime and disorder might increase inequalities in health [24] through feelings of discrimination, and stress [25, 65]. Conversely, neighborhood collective efficacy and social cohesion [57] might mitigate stress and reduce mental health problems by providing social support [65]. Therefore, neighborhood-level factors could result in inequalities in anxiety in perinatal minority women above and beyond individual-level factors.

Regarding mechanisms that connect neighborhood disorder and health, previous research points to neighborhood environmental stressors as a key pathway to poor health and health inequalities. Stressors included urbanization, low SES, overcrowding, and low social cohesion and social support [7, 69]. For example, Generaal et al. [27] found high prevalence and increased severity of depression and anxiety in low-SES neighborhoods [27]. Regardless of individual-level income, neighborhood low SES, neighborhood low income level [5, 39, 40], and neighborhood deprivation [29] have all been associated with depressive disorders, major depressive episodes [5, 39, 40], depressive symptoms and anxiety disorders [56, 71]. The current examines in the associations between neighborhood violence and disorder and inequalities in anxiety among perinatal Jewish women Palestinian-Arab minority in Israel living in segregated neighborhoods.

### Residential segregation and neighborhood violence and disorder in Israel

The ethno-national divide between the Palestinian-Arab minority (about one fifth of citizens) and Jewish majority populations in Israel has deep historical and political roots that have brought about ethno-national residential segregation, which might affect health. These groups live almost fully in separate geographic areas, local authorities and neighborhoods that are divided along ethno-national lines [37]. The establishment of the Jewish state in 1948 and the prolonged Arab-Jewish conflict that followed left the Palestinian-Arab minority subordinate to the Jewish majority [37]. This inequality persists today in multiple areas of life, including the labor market [37, 41, 50]; education [1]; housing [66]; and healthcare [16]. Palestinian-Arab villages have also seen rapid urbanization in recent decades [33], with their enclave economies transformed from agricultural to semi-industrial and service based [37]. Discriminatory governmental policies, confiscation of lands and low resource allocation towards Palestinian-Arab local authorities and neighborhoods have led to systematic deprivation [18, 47]; lower SES [47, 50]; and, in recent years, to unprecedented street violence and crime [15]. These changes have been shown to increase neighborhood problems and erode collective efficacy (social cohesion, informal social control) and social capital in Palestinian-Arab neighborhoods [15], two factors that were associated with higher violence against women, including intimate partner violence (IPV) [11]. All of these factors affect health [18]. Previous research showed higher levels of mental health problems in the Palestinian-Arab population compared to Jewish Israelis [45], including for women. For example, postpartum depression was higher among Palestinian-Arab minority mothers compared to Jewish mothers [60]. However, no studies compared anxiety in these population groups. More than a decade ago one study examined the prevalence of anxiety in Israel’s general population and found low levels of mood and anxiety disorders [46]. We know of no studies that considered links between neighborhood environment, including neighborhood violence and disorder, and health inequalities between these population groups in Israel. The current study aims to examine the association between neighborhood violence and disorder and inequalities in anxiety between two groups of perinatal Israeli women: Jewish and Palestinian-Arab minority living in ethno-nationally segregated neighborhoods (low socioeconomic status (SES) neighborhoods, neighborhoods characterized by low collective efficacy including social cohesion and informal social control, and low social capital, and high aggregate discrimination; while considering social support and chronic stress as important factors that might influence this association..

### Study framework

Our conceptual framework draws on Diez & Mair’s framework for residential segregation and health inequalities [24]. Diez & Mair [24] proposed two interrelated processes for residential segregation that might impact resident behaviors and stress and contribute to inequalities in health. These include neighborhood physical attributes, such as the neighborhood built environment, environmental exposures, SES, services, and resources; and neighborhood social attributes, including neighborhood social cohesion, collective efficacy, and social capital. Physical and social attributes may interact to produce ill health amidst residential segregation [24].

We suggest that ethno-national inequalities in anxiety in perinatal women will be associated with inequalities in neighborhood violence and disorder regardless of individual-level attributes. Further, we propose that, in looking at our sample, we will see the workings of residential segregation and an ethno-national divide, which might play important roles as mechanisms for health disparities within and between Israel’s largest ethnic-national groups [14]. We assume that we will find higher neighborhood violence and disorder in Palestinian-Arab women’s neighborhoods, as these suffer from prolonged policies of discrimination stemming from segregation. Residential segregation will be manifested by lower SES, higher aggregate discrimination, and lower collective efficacy (social cohesion and informal social control), low social capital in Palestinian-Arab women’s neighborhoods compared to Jewish women’s neighborhoods. Taken together, these factors together with low social support will be associated with elevated stress in Palestinian-Arab women and be associated with inequalities in anxiety in Palestinian-Arab perinatal women compared to Jewish women. Thus, we put forward the following hypotheses:

1. Compared to Jewish women, Palestinian-Arab women will report higher neighborhood violence and disorder and higher anxiety, as they live in neighborhoods that suffer from prolonged residential segregation, and based on three aspects of neighborhood disadvantage: low SES [37, 66], high levels of neighborhood aggregate discrimination [17] and low neighborhood social factors (low collective efficacy (social cohesion and informal social control) and low social capital, [15].
2. Neighborhood crime and disorder will be associated with higher anxiety in all women, but this association will be stronger among Palestinian-Arab women compared to Jewish women, as Palestinian-Arab women will present increased levels of chronic stress and low social support resulting from more neighborhood disadvantaged including lower levels of collective efficacy (social cohesion and informal control), lower social capital, higher neighborhood economic deprivation [18], as well as higher neighborhood aggregate discrimination) [19].

## Methods

### Study design and data

We obtained data for the current analysis from the “Family Relations, Violence and Health” study which we linked to census data on neighborhood SES. The survey was conducted from October 2014 to April 2015 and included a stratified cluster sample of perinatal Palestinian-Arab and Jewish women citizens of Israel who were interviewed while visiting a sample of Maternal and Child Health (MCH) clinics. Nearly all mothers in Israel visit MCH clinics after birth for well-baby follow-up care and immunizations. The MCH clinics in the study were located in 63 neighborhoods in 5 regions of the country that represent different ethno-national and socioeconomic groups of mothers [20]. The number of MCH clinics and women in each district was proportional to the number of births in a district and the ethno-national compositions of the women. Each MCH clinic is located in a different neighborhood and forms one cluster in the study. A detailed description of the study sample and sampling procedure are presented elsewhere [20], but briefly, the study included a stratified cluster sample of 1401 women (Palestinian-Arab = 436, Jewish = 965) who were recruited while visiting MCH clinics (response rate: 75%). After the clinics were selected in each district, perinatal women who visited these clinics for pregnancy follow-up, well-baby care and immunizations were recruited for the study. Recruitment was as follows: upon arrival at the clinics, eligible women (pregnant or postpartum, 6 weeks to 6 months after birth) were approached by trained female interviewers who asked them about participating in the study. Women who agreed signed an informed consent form and were invited to a separate room at the clinic for a private 30–45–minute interview conducted in their native language (Arabic or Hebrew). Women who showed high symptoms of anxiety or depression were referred to nurses and received contact information for local social and health support services.

### Study measures

***Anxiety*** was measured by 10 items from the “State-Trait Anxiety Inventory” (STAI) [62, 63], including: I feel calm, I feel secure, I feel tense, I feel strained, I feel at ease, I feel upset, I’m presently worrying over possible misfortunes, I feel satisfied, I feel frightened, I feel uncomfortable,” with four answer categories ranging from ‘never’ to ‘almost always.’ These items were used in previous study in Israel [17]. We used reverse coding for positively worded items and summed the answers to create a total score, then dichotomized this score using the median score as a cutoff. Low anxiety <median vs. high anxiety ≥median score. Notably, the STAI is not a diagnostic clinical tool, but rather, a tool to measure the presence of anxiety symptoms. The reliability test revealed Cronbach’s alpha of 0.76 for Jewish women, 0.74 for Palestinian-Arab women.

#### Neighborhood level measures

Neighborhood measures in the current study were aggregate variables, or variables measured by questions that ask about the neighborhood, as described below for each variable.

***Neighborhood crime and disorder*** was measured based on a previous questionnaire [61]. We asked 10 questions about neighbourhood violence and disorder. These include for example abandoned buildings, vandalism, shootings, murder in the street, unsafe feelings while walking at night, etc. Women who answered in the affirmative (yes) to the presence of these problems in their neighbourhood were asked to rate these problem’s severity. The rating included a Likert scale of four categories ranging from ‘not at all’ to ‘very severe.’ We calculated the sum score of positive answers and dichotomized responses at the median score of neighbourhood violence and disorder into: high≥ median and low <median, as the distribution of the sum score was asymmetrical. Then we combined the two variables of presence and severity of neighbourhood violence and disorder and created a new variable of neighbourhood violence and disorder that included three answer categories: no problem, low disorder, and high disorder. Cronbach’s alpha for this variable was 0.98.

***Neighborhood socioeconomic status (SES)*** was obtained from census data collected by Israel’s Central Bureau of Statistics (CBS) [10]. The census defines a neighborhood as a geographic-statistical unit or area composed of 5000 residents (some Palestinian-Arab villages are of this size). The CBS calculates the socioeconomic status (SES) of each geographical-statistical area based on factor analysis for groups of variables, including sociodemographic variables, and socioeconomic variables such as education, employment and income, and standard of living variables [11]. The standardized sum score of all variables ranges from 1 to 20, where 1 is the lowest for each of the statistical units, 20 the highest. In our study we linked the census data on neighborhood SES to each participating women’s data based on her address in a neighborhood. In cases of missing home addresses, we substituted a neighborhood MCH clinic address, as women attend the clinic located in their neighborhood. We divided the CBS neighbourhood SES index,, into 3 levels: low for neighborhoods with the lowest SES index of 1–5; medium for a SES index of 6–9; and high for neighborhoods with a SES index of 10–18 (as 18 was the upper limit in the current study).

***Neighborhood social characteristics:*** These included two measures of collective efficacy, two measures of social capital [58], and one measure of aggregate discrimination Collective efficacy was measured by *social cohesion* and *informal social control*. Social cohesion was measured based on participants’ level of agreement (agree very much, agree, and do not agree) with 5 statements on neighborhood social networks, mutual help, and trust [58]. For example, ‘This is a close-knit neighborhood.’ We reversed the score of the last two statements and dichotomized the sum score at the median (high social cohesion≥ median, and low social cohesion<median) as the total score was not symmetrical. Cronbach’s alpha was 0.72 for Jewish women, 0.71 Palestinian-Arab women.

*Informal social control* was based on participants’ level of agreement (ranging from ‘agree very much’ to ‘do not agree at all’) with four statements, which we adapted to the Israeli context from Sampson et al. [58]. The statements examine neighbors’ willingness to intervene regarding local problems such as: ‘children skipping school and hanging out on a street corner’; ‘a fight breaking out nearby’; ‘children showing disrespect to an adult’; and ‘problems with the water or electricity.’ We calculated a sum score, which we dichotomized at the median score into: high (≥median) and low (<median) informal social control, as the distribution of the total score was asymmetrical. Cronbach’s alpha was 0.79 for Jewish women, 0.75 for Palestinian-Arab women.

The third and fourth neighborhood social attributes included bridging and linking social capital. Bridging social capital was measured by *social group membership,* which included 8 yes/no questions on membership in community groups or organizations in the last 12 months: ‘work-related/trade union’; ‘religious group’; ‘women’s group’; ‘sport group’; ‘political organization’; ‘ethnic group organization’ and ‘neighborhood committee’ [21, 38]. We dichotomized the sum of responses into: ‘membership in at least one group’ and ‘no group membership.’ Linking social capital included 7 yes/no questions about *participation in social or political activities* in the last 12 months [30], such as, “Have you participated in a meeting of a neighborhood committee to discuss problems in your community?” and, “Have you talked with the media about problems in your community?” We dichotomized responses into: participated at least one time, and never participated.

The fifth measure of neighborhood social attributes was aggregate discrimination. This variable was created by calculating the percentage of women in a neighborhood who reported feeling discriminated against because of their ethno-national identity. The distribution of the aggregate discrimination scale was asymmetric, and we have dichotomized this variable into ‘high aggregate discrimination’ (>median score) and ‘low aggregate discrimination’ (≤median). The median score used for this cut-off was 26.09%.

**Individual-level control variables** included *sociodemographic variables*: age (16–24, 25–34 and 35–48 years), country of birth (Israel or other) and marital status (1. Married; and 2. not married including: single, divorced, separated, not-cohabitating, or other). *Women’s socioeconomic status* included women’s education (high school or less; college education but not university; and bachelor’s or higher education); employment (work or not outside the household); and family income (work only; social allowances only or other, including any combination of work and social benefits, or family and/or a friend support). *Women’s obstetrics history* was measured by women’s status during the interview, which we arrived at by combining pregnancy status and having other children (pregnant with no children; pregnant with children; after birth with 1–2 children; after birth with 3 or more children). Finally, *previous depression* was determined by using a yes /no question on using prescribed antidepressants and diagnosed with depression before the most recent pregnancy. We considered this variable a proxy for previously diagnosed depression among the women.

#### Chronic stress and social support

In the current study chronic stress and social support were assumed to play a role as possible mediator. Chronic stress was measured by the sum score of responses to 10 yes/no questions on past experiences with stressful situations (e.g., financial, social, family, or work problems) [64]. Because of asymmetric distribution we dichotomized the sum score (ranging from 0 to 9) based on the median score (median = 0); ‘no stress’ = median score and ‘any stress’ > median. Since chronic stress had 11% missing data and the women who had missing data for chronic stress had high anxiety (54%), we decided to create a third category called ‘missing data’ to allow those women to be part of further analysis. Thus, the categories for the variable of chronic stress were: any stress, low stress and missing data.

Social support was measured as the sum score of responses to a 6-item scale encompassing three types of support—material, emotional and informational—with 6 response categories ranging from never to always [35]. We dichotomized answer scores ranging from 1 to 5 at the median score of 4.83 (SD = 0.81) as the distribution of the scale was asymmetric. We assigned a score ≤ median as ‘low social support,’ and > median as ‘high social support.’ Cronbach’s alpha was 0.89 for the total sample, and 0.86 and 0.87 for Jewish and Palestinian-Arab women, respectively.

### Data analysis

We conducted multivariable logistic regression analysis using Generalized Estimation Equation (GEE) to estimate the associations (odds ratios) between neighborhood violence and disorder and anxiety in the total sample and for each ethno-national group of women, who live in segregated neighborhoods. Since ours was a cluster sample and each of the Maternal and Child Health Clinics (MCH) forms a cluster, we used the GEE procedure to account for the inter-cluster effect. The GEE analysis was conducted after we examined the univariate associations (using *X*^*2*^ test) between all independent individual-level and neighborhood-level variables and anxiety. We excluded from the GEE analysis variables that were not associated with anxiety in the univariate analysis (*P* > 0.05).

Notably, the missing data was less than 10% for all variables except chronic stress, which had high missing cases (11.6%). Since we found that this group of women had different characteristics from women who reported chronic stress, we decided to keep, as a separate category of chronic stress women who had missing data for chronic stress (as described above). To estimate inequalities in anxiety between the two ethno-national groups of women first we conducted GEE analysis in 4 multivariable logistic regression models for the association between neighborhood violence and disorder and anxiety for the total sample and then separately for each ethno-national group. For the total sample we compared the OR of the associations between neighborhood violence and disorder anxiety while considering women’s ethno-national group Palestinian-Arab and Jewish women and conducting the following adjustments in different models: Model 1- included the unadjusted association between neighborhood violence and disorder, and anxiety; Model 2- adjusted for ethno-national identity and individual level variables (taking anti-depressants, and women’s employment) in addition to variables in Model 1; Model 3- added adjustments for neighborhood-level variables of social cohesion, social capital, SES, and aggregate discrimination; Model 4- considered social support in addition to variables in Model 3, and Model 5 adjusted for all variables in Model 4 in addition to considering chronic stress. To explore factors that contributed to inequalities in the association between neighborhood attributes and anxiety in each of the ethno-national groups, we conducted GEE analysis for each group of women while repeating the same Models (1–5), but without considering ethno-national identity.

## Results

**Fig. 1:**
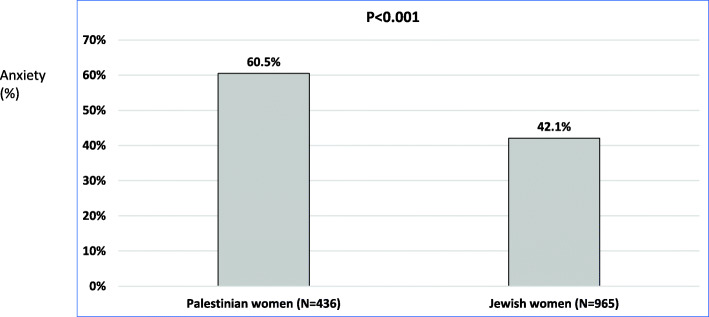
Prevalence of anxiety (%) among Palestinian-Arab and Jewish women in the study sample

**Fig. 2:**
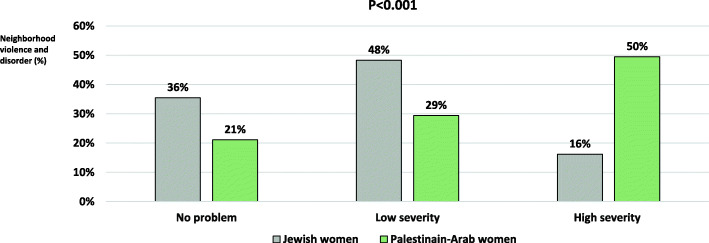
Neighborhood violence and disorder (%) among Palestinian-Arab and Jewish women in the study sample

The current study included a sample of 1401 women, of whom two thirds (*N* = 963) were Jewish and the rest (*N* = 436) minority Palestinian-Arab. Compared to Jewish women, Palestinian-Arab women had higher prevalence of anxiety (60.5, 95% Confidence intervals (CI) = 55.7–65.1% vs. 42.1% (95%CI = 38.9–45.3%), respectively) (Fig. 1). Palestinian-Arab women reported higher neighborhood problems in average (mean, SD = 3.18, 2.92; 95%CI = 1.68–1.93) compared to Jewish women (mean, SD = 1.81, 2.02; 95%CI = 2.91–3.46). Also, Palestinian-Arab women reported greater severity of neighborhood violence and disorder than Jewish women. High severity of neighborhood violence and disorder was 49.5% (95%CI = 44.8–54.3%) among Palestinian-Arab vs. 16.2% (95%CI = 13.9–18.6%) among Jewish women (Fig. 2).

The two ethno-national groups had significantly different neighborhood- and individual-level characteristics (Table 1). Compared to Jewish women, Palestinian-Arab women lived in neighborhoods characterized by lower collective efficacy (lower social cohesion and informal social control); lower social capital (lower social-group membership and social and political participation); lower neighborhood SES; and higher ethno-national neighborhood aggregate discrimination (Table 1).

**Table 1:** Individual and neighborhood characteristics of Palestinian-Arab and Jewish women in the study sample and associations with anxiety

	Total	Jewish (***N*** = 965) 68.9%	Arab (***N*** = 436) 31.1%	***P***^**a**^	Anxiety (***N*** = 666) 47.8%	***P***^**a**^
	N	N (%)	N (%)		N (%)	
**Age**				< 0.001		0.079
35–48	309	257 (26.6)	52 (12.0)		140 (45.8)	
25–34	844	624 (64.7)	220 (50.6)		398 (46.7)	
16–24	247	84 (8.7)	163 (37.5)		133 (54.3)	
**Country of birth**				< 0.001		0.827
Israel	1133	722 (75.3)	411 (94.9)		534 (47.5)	
Other	259	237 (24.7)	22 (5.1)		125 (48.3)	
**Marital status**				< 0.001		0.463
Married	1329	901 (93.6)	428 (98.8)		629 (47.7)	
Unmarried	67	62 (6.4)	5 (1.2)		35 (52.2)	
**Women’s status**				< 0.001		0.622
Not pregnant with ≥3 children	390	282 (29.3)	108 (24.9)		181 (46.5)	
Not pregnant with 1–2 children	737	569 (59.2)	168 (38.8)		350 (47.5)	
Pregnant with children	187	82 (8.5)	105 (24.2)		91 (50.0)	
Pregnant without children	80	28 (2.9)	52 (12.0)		42 (53.8)	
**Education**				< 0.001		0.095
Less than high school	537	259 (26.8)	278 (63.8)		274 (51.4)	
College and beyond high school	251	188 (19.5)	63 (14.4)		116 (47.2)	
Bachelor and higher	613	518 (53.7)	95 (21.8)		276 (45.0)	
**Current employment**				< 0.001		< 0.001
Yes	785	675 (71.0)	110 (25.3)		337 (43.4)	
No	600	276 (29.0)	324 (74.7)		324 (54.1)	
**Family income**				< 0.001		0.154
From work	982	642 (66.5)	340 (78.0)		467 (47.8)	
Social allowances	79	26 (2.7)	53 (12.2)		45 (57..7)	
Work and another source	340	297 (30.8)	43 (9.9)		154 (45.6)	
**Taking antidepressants**				0.076		0.023
No	1364	941 (98.2)	423 (99.5)		645 (47.4)	
Yes	19	17 (1.8)	2 (0.5)		14 (73.7)	
**Chronic stress**				< 0.001		< 0.001
No	629	454 (47.0)	175 (40.1)		229 (36.4)	
Any	610	442 (45.8)	168 (38.5)		354 (58.0)	
Missing	162	69 (7.2)	93 (21.3)		83 (54.2)	
**Social support**				< 0.001		< 0.001
Low	613	305 (31.8)	308 (71.1)		372 (61.0)	
High	780	655 (68.2)	125 (28.9)		292 (37.6)	
**Neighborhood SES**				< 0.001		< 0.001
Low	407	50 (5.2)	357 (82.1)		239 (46.6)	
Medium	471	397 (41.4)	74 (17.0)		195 (41.5)	
High	516	512 (53.4)	4 (0.9)		232 (57.1)	
**Neighborhood aggregate discrimination**				< 0.001		< 0.001
Low	708	577 (65.0)	131 (30.3)		306 (43.5)	
High	612	311 (35.0)	301 (69.7)		330 (54.0)	
**Social cohesion**				< 0.001		< 0.001
Low	911	594 (62.0)	317 (73.2)		476 (52.7)	
High	480	364 (38.0)	116 (26.8)		188 (39.3)	
**Informal social control**				< 0.001		0.049
Low	866	521 (54.2)	345 (79.5)		429 (49.9)	
High	530	441 (45.8)	89 (20.5)		235 (44.5)	
**Social group membership**				< 0.001		0.968
No membership	915	581 (60.3)	334 (76.8)		435 (47.8)	
At least one group membership	484	383 (39.7)	101 (23.2)		230 (47.9)	
**Social and political participation**				< 0.001		0.041
Never participated	788	435 (45.1)	353 (81.1)		393 (50.3)	
Participated at least once	611	529 (54.9)	82 (18.9)		272 (44.7)	

^a^Chi square test

Regarding individual-level characteristics, Table 1 shows that Palestinian-Arab women were more young, born in Israel, married, and pregnant with or without previous children at the time of the interview. Additionally, there were important differences between Jewish and Palestinian-Arab women’s SES: Jewish women had significantly higher education and family income and were more employed at the time of the interview. We found no significant differences regarding taking anti-depressants. Apparently, Palestinian-Arab women had lower levels of chronic stress and lower social support compared to Jewish women. However Palestinian-Arab women also presented more missing cases for chronic stress than Jewish women. (Table 1).

Table 1 also shows univariate associations between study variables and anxiety. Unemployed women, those taking anti-depressants, with any chronic stress, and those with lower social support reported higher anxiety. However, we found no significant associations between the other sociodemographic variables (age, country of birth, marital status, women’s status at time of interview, education) and anxiety (Table 1).

Regarding neighborhood characteristics, in the univariate associations (Table 1) we found higher levels of anxiety among women living in lower collective efficacy neighborhoods (lower social cohesion and informal social control) and lower linking social capital neighborhoods (lower social and political participation). Also, women living in lower SES neighborhoods, and in neighborhoods that had higher levels of aggregate ethno-national discrimination had higher levels of anxiety (Table 1).

Table 2 shows the distribution of 10 forms of neighborhood violence and disorder, and the total score for all ten forms by ethno-national identity, and associations with anxiety. Palestinian-Arab women consistently reported higher neighborhood violence and disorder for each of the 10 forms of neighborhood violence and disorder and for the total score. Women who reported higher severity of neighborhood violence and disorder also had higher anxiety (Table 2).

**Table 2:** Neighborhood violence and disorder among Palestinian-Arab and Jewish women and associations with anxiety

Neighborhood violence and disorder	Total	Jewish (***N*** = 965) 68.9%	Arab (***N*** = 436) 31.1%	***P***^a^	Anxiety (***N*** = 666) 47.8%	P^a^
	N	N (%)	N (%)			
**1. Garbage in the street or on the sidewalk**				< 0.001		0.003
No	732	556 (57.7)	176 (40.5)		321 (44.0)	
Yes	667	408 (42.3)	259 (59.5)		344 (52.0)	
**2. Empty buildings or abandoned cars**	11,061	802		< 0.001		0.003
No	1106	802 (83.2)	304 (70.0)		503 (45.9)	
Yes	292	162 (16.8)	130 (30.0)		162 (55.5)	
**3. Neglected or missing sidewalks**	974			< 0.001		0.021
No	974 4 24	730 (75.7) 234	244 (56.2)		442 (45.8)	
Yes	424	234 (24.3)	190 (43.8)		222 (52.5)	
**4. Loitering of drug dealers or criminals**				< 0.001		0.019
No	1192 202	860 (89.4) 102	332 (76.9) 100		553 (46.6)	
Yes	202	102 (10.6)	100 (23.1)		110 (55.6)	
**5. Groups of children, youth or adults who act improperly or wildly**				< 0.001		< 0.001
No	910	663 (68.8)	247 (56.8)		400 (44.3)	
Yes	489	301 (31.2)	188 (43.2)		265 (54.4)	
**6. Vandalism or property damage**		757		< 0.001		< 0.001
No	1044 354	575 (78.5) 207	287 (66.1) 14		467 (45.0)	
Yes	354	207 (21.5)	147 (33.9)		197 (56.1)	
**7. Drug use or overuse of alcohol**				< 0.001		0.003
No	1206 191	854 (88.7) 109 (	352 (81.1) 82		554 (46.2)	
Yes	191	109 (11.3)	82 (18.9)		109 (57.7)	
**8. Police harassment**	9			< 0.001		0.012
No	1320 78	924 (96.0) 39	396 (91.0) 39		616 (47.0)	
Yes	78	39 (4.0)	39 (9.0)		48 (61.5)	
**9. Shooting or murder of people on the street**				< 0.001		< 0.001
No	1224 175	917 (95.1) 4	307 (70.6)		557 (45.8)	
Yes	175	47 (4.9)	128 (29.4)		108 (61.7)	
**10. People scared to walk on the street at night**				< 0.001		< 0.001
No	1062 257	812 (85.7)	250 (67.2)		471 (44.4)	
Yes	257	135 (14.3)	122 (32.8)		144 (56.3)	
**Neighborhood violence and disorder (total)**				< 0.001		< 0.001
No problem	435	343 (35.5)	92 (21.1)		168 (38.9)	
Low severity	594	466 (48.3)	128 (29.4)		298 (50.2)	
High severity	379	156 (16.2)	46 (49.5)		200 (54.6)	

^a^Chi-square test

It is important to identify the characteristics of women in the total sample who reported higher severity of neighborhood violence and disorder (Table 3). These women were younger (16-24 yrs), with lower education (less than high school), more often unemployed, had family income more often coming from social allowances, and reported less taking anti-depressants. They also reported lower social support and higher levels of any chronic stress, and had missing values of chronic stress. Regarding neighborhood attributes, higher neighborhood violence and disorder was reported by women living in neighborhoods characterized by low (vs. high) collective efficacy (social cohesion and informal social control); and lower linking and bridging social capital, as defined by low (vs. high) social and political participation, low (vs. high) social-group membership, and high (vs. low) aggregate ethno-national discrimination (Table 3). Notably, country of birth, marital status and women’s status during the interview had no significant associations with neighborhood violence and disorder.

**Table 3:** Univariate associations between individual and neighborhood characteristics and neighborhood violence and disorder (Total *N* = 1401)

	Total	No problem	Low severity	High severity	***P***^a^
	N (%)	N (%)	N (%)	N (%)	
**Age**					< 0.001
35–48	309 (22.1)	95 (30.7)	144 (46.6)	70 (22.7)	
25–34	844 (60.3)	268 (31.8)	374 (44.3)	202 (23.9)	
16–24	247 (17.6)	71 (28.7)	76 (30.8)	100 (40.5)	
**Country of birth**					0.713
Israel	1133 (81.4)	354 (31.2)	473 (41.7)	306 (27.0)	
Other	259 (18.6)	79 (30.5)	115 (44.4)	65 (25.1)	
**Marital status**					0.703
Married	1329 (95.2)	411 (30.9)	562 (42.3)	356 (26.8)	
Unmarried	67 (4.8)	21 (31.3)	31 (46.3)	15 (22.4)	
**Women’s status**					0.093
Pregnant without children	80 (5.7)	26 (32.5)	28 (35.0)	26 (32.5)	
Pregnant with children	187 (13.4)	52 (27.8)	72 (38.5)	63 (33.7)	
Not pregnant with 1–2 children	737 (52.9)	236 (32.0)	328 (44.5)	173 (23.5)	
Not pregnant with ≥3 children	390 (28.0)	121 (31.0)	162 (41.5)	107 (27.4)	
**Education**					< 0.001
Bachelor and higher	425 (30.3)	139 (32.7)	205 (48.2)	81 (19.1)	
Beyond high college	241 (17.2)	86 (35.7)	97 (40.2)	58 (24.1)	
Less than high school	537 (38.3)	142 (26.4)	190 (35.4)	205 (38.2)	
**Current Employment**					< 0.001
Yes	785 (56.7)	275 (35.0)	362 (46.1)	148 (18.9)	
No	600 (43.3)	155 (25.8)	223 (37.2)	222 (37.0)	
**Family income**					0.004
From work	982 (70.1)	314 (32.0)	402 (40.9)	266 (27.1)	
Social allowances	79 (5.6)	21 (26.6)	26 (32.9)	32 (40.5)	
Work and another source	340(24.3)	100 (29.4)	166 (48.8)	74 (21.8)	
**Taking antidepressants**					0.049
No	1364 (98.6)	425 (31.2)	585 (42.9)	354 (26)	
Yes	19 (1.4)	7 (36.8)	8 (42.1)	4 (21.1)	
**Chronic stress**					< 0.001
No	629 (44.9)	250 (39.7)	233 (37.0)	146 (23.2)	
Any	610 (43.5)	143 (23.4)	292 (47.9)	175 (28.7)	
Missing	162 (11.6)	42 (25.9)	69 (42.6)	51 (31.5)	
**Social support**					< 0.001
Low	613 (44.0)	145 (23.7)	266 (43.4)	202 (33.0)	
High	780 (56.0)	287 (36.8)	326 (41.8)	167 (21.4)	
**Neighborhood SES**					< 0.001
Low	407 (29.2)	93 (22.9)	118 (29.0)	196 (48.2)	
Medium	471 (33.8)	140 (29.7)	219 (46.5)	112 (23.8)	
High	516 (37.0)	199 (38.6)	256 (49.6)	61 (11.8)	
**Neighborhood aggregate discrimination**					< 0.001
Low	708 (53.6)	259 (36.6)	302 (42.7)	147 (20.8)	
High	612 (46.4)	144 (23.5)	251 (41.0)	217 (35.5)	
**Social cohesion**					< 0.001
Low	911 (65.5)	249 (27.3)	391 (42.9)	271 (29.7)	
High	480 (34.5)	181 (37.7)	200 (41.7)	99 (20.6)	
**Informal social control**					< 0.001
Low	866 (62.0)	236 (27.3)	360 (41.6)	270 (31.2)	
High	530 (38.0)	196 (37.0)	233 (44.0)	101 (19.1)	
**Social group membership**					0.013
No membership	915 (65.4)	301 (32.9)	363 (39.7)	251 (27.4)	
At least one group membership	484 (34.6)	133 (27.5)	231 (47.7)	120 (24.8)	
**Social and political participation**					< 0.001
Never participated	788 (56.3)	269 (34.1)	287 (36.4)	232 (29.4)	
Participated at least once	611 (43.7)	165 (27.0)	307 (50.2)	139 (22.7)	

^a^Chi-square test

Results of the multi-variable GEE analysis for the total sample (Table 4) showed that high (vs. no) neighborhood violence and disorder was consistently associated with higher anxiety in all models, and this association became insignificant for high severity only after adjustment for chronic stress in the fully adjusted model (Model 5). Model 1 showed that anxiety was almost twice as high among women living in neighborhoods with high (vs. no) severity of violence and disorder (crude odds ratio (OR) = 1.83, 95%CI = 1.31–2.57).

**Table 4:** Generalized Estimating Equation (GEE) for the association between neighborhood violence and disorder and anxiety among the total sample of perinatal women in the study (*N* = 1270)

	Model 1	Model 2	Model 3	Model 4	Model 5
	OR (95%CI)	OR (95%CI)	OR (95%CI)	95%CI	95%CI
**Neighborhood violence and disorder**					
High severity	1.83 (1.31.-2.57)^***^	1.44 (1.08–1.91)^*^	1.43 (1.08–1.89)**	1.39 (1.04–1.86)**	1.25 (0.91–1.71)
Low severity	1.54 (1.21–1.96)^***^	1.56 (1.23–1.97)^***^	1.50 (1.19–1.89)***	1.41 (1.11–1.79)***	1.28 (1.00–1.64)**
No problem	1.00	1.00	1.00	1.00	1.00
**Ethno-national identity**					
Palestinian-Arab		1.82 (1.23–2.69)^**^	2.12 (1.34–3.34)***	1.68 (1.09–2.60)**	1.63 (1.04–2.56)**
Jewish		**1.00**	**1.00**	**1.00**	1.00
**Neighborhood SES**					
Low			0.57 (0.35–0.93)*	0.56 (0.36–0.90)*	0.60 (0.37–0.99)*
Medium			0.62 (0.41–0.93)*	0.61 (0.40–0.92)*	0.61 (0.40–0.93)*
High			**1.00**	**1.00**	1.00
**Neighborhood aggregate discrimination**					
High			1.30 (0.92–1.84)	1.27 (0.91–1.76)	1.25 (0.86–1.81)
Low			1.00	1.00	1.00
**Social cohesion**					
High			0.62 (0.49–0.77)***	0.66 (0.52–0.83)***	0.65 (0.52–0.83)***
Low			**1.00**	**1.00**	**1.00**
**Social group membership**					
At least one group membership			1.05 (0.83–1.33)	1.07 (0.84–1.35)	1.06 (0.84–1.34)
No membership			1.00	1.00	1.00
**Social and political participation**					
Participated at least once			0.93 (0.69–1.26)	0.94 (0.70–1.26)	0.83 (0.61–1.14)
Never participated			1.00	1.00	1.00
**Social support**					
High				0.47 (0.38–0.58)***	0.50 (0.40–0.63)***
Low				**1.00**	**1.00**
**Chronic Stress**					
Missing					1.43 (0.86–2.37)
Any					**2.20 (1.75–2.77)** ***
No					**1.00**
Model fit	1746.30	1734.86	1703.19	1707.74	1639.57

***P ≤ 0.001; **P ≤ 0.010; *P ≤ 0.050 (bold = statistically significant associations)

Model 1 is unadjusted;

Model 2 controls for ethno-national group and individual-level attributes (previous depression and unemployment);

Model 3 controls for ethno-national group, individual-level attributes and neighbohood-level characteristics;

Model 4 adjusts for social support in addition to variables in Model 3

Model 4 adjusts for chronic stress in addition to variables in Model 4

Adjustment for ethno-national identity in Model 2 (in addition to individual-level attributes) was associated with attenuation of the association (OR) between neighborhood violence and disorder and anxiety by almost 20% (Table 4). In Model 2 ethno-national identity was associated with almost twofold anxiety among Palestinian-Arab women compared to Jewish women (AOR, 95%CI = 1.82,1.24–2.68) and this association was significant also in the fully adjusted model (AOR, 95%CI = 1.63, 1.04–2.56) (Table 4, Model 5).

Adjusting for neighborhood attributes beyond individual level factors in Model 3 (Table 4) did not change much the OR of the association between neighborhood violence and disorder and anxiety . and this association was still significant. High SES neighborhoods, high social cohesion and high social support were protective factors from anxiety. In Model 4, adjusting for social support in addition to individual level and neighborhood level factors reduced the OR only a little compared to Models 2 and 3. Only in Model 5 we noticed significant reduction in the OR. Adjusting for chronic stress resulted in non-significance association between high severity of neighborhood violence and disorder and anxiety as mentioned above (Table 4).

To explore the factors related to neighborhood violence and disorder that contribute to anxiety in each ethno-national group of women we conducted separate multi-level analyses for Jewish women and for Arab women (Table 5). In Jewish women (Table 5) the likelihood of anxiety was higher among women who reported low neighborhood violence and disorder compared to Jewish women who reported no neighborhood disorder (Crude OR = 1.70, 95%CI = 1.30–2.23). The category of high severity of neighborhood violence and disorder had no significant association with anxiety in Jewish women. Adjustment for other individual level and neighborhood-level variables and social support in Models 2–4 showed that the association between neighborhood violence and disorder and anxiety did not change much. Only when adjusting for chronic stress in the fully adjusted model (Model 5) the OR of the association between neighborhood disorder and anxiety was attenuated by 20%, but the association remained significant for low severity (AOR, 95%CI = 1.41, 1.07–1.86). Chronic stress was a risk factor for anxiety in Jewish women (AOR = 2.04, 95%CI = 1.54–2.71). High-SES neighborhoods, high social-cohesion neighborhoods and social support were protective factors from anxiety in Model 5 in Jewish women (Table 5).

**Table 5:** Generalized Estimating Equation (GEE) results for the association between neighborhood violence and disorder, and anxiety among Jewish women (*N* = 860)

	Model 1	Model 2	Model 3	Model 4	Model 5
	OR (95%CI)	OR (95%CI)	OR (95%CI)	OR (95%CI)	OR (95%CI)
**Neighborhood disorder**					
High severity	1.16 (0.80–1.68)	1.16 (0.79–1.69	1.22 (0.81–1.84)	1.22 (0.82–1.85)	1.00 (0.66–1.53)
Low severity	1.70 (1.30–2.23)^***^	1.72 (1.32–2.25)^***^	1.69 (1.30–2.20)***	1.69 (1.30–2.20)^***^	1.41 (1.07–1.86)^**^
No problem	1.00	1.00	1.00	1.00	1.00
**Neighborhood SES**					
Low			0.54 (0.29–1.03)	0.52 (0.26–1.01)	0.53 (0.30–0.94)^**^
Medium			0.67 (0.44–1.04)	0.66 (0.43–1.01)	0.67 (0.44–1.01)
High			1.00	1.00	1.00
**Neighborhood aggregate discrimination**					
High			1.11 (0.70–1.74)	1.10 (0.70–1.73)	1.01 (0.66–1.55)
Low			1.00	1.00	1.00
**Social cohesion**					
High			0.70 (0.55–0.89)**	0.70 (0.54–0.89)^**^	0.75 (0.58–0.98)^**^
Low			**1.00**	**1.00**	**1.00**
**Social group membership**					
At least one group membership			1.02 (0.78–1.34)	1.00 (0.75–1.33)	0.96 (0.73–1.27)
No membership			1.00	1.00	1.00
**Social and political participation**					
Participated at least once			0.91 (0.66–1.25)	0.91 (0.66–1.26)	0.81 (0.58–1.13)
Never participated			1.00	1.00	1.00
**Social support**					
High				0.50 (0.38–0.65)^***^	0.51 (0.39–0.68)^***^
Low				1.00	**1.00**
**Chronic Stress**					
Missing					1.36 (0.63–2.93)
Any					2.04 (1.54–2.71)^***^
No					1.00
Model fit	1169.97	1167.85	1164.67	1164.67	1126.17

***P ≤ 0.001; **P ≤ 0.010; *P ≤ 0.050 (bold = statistically significant associations). Model 1 is unadjusted;

Model 2 controls for individual-level attributes (previous depression and unemployment);

Model 3 controls for individual-level attributes and neighbohood-level characteristics;

Model 4 adjusts for social support in addition to variables in Model 3.Model 5 adjusts for chronic stress in addition to variables in Model 4

In Palestinian-Arab women (Table 6) the likelihood of anxiety was higher among women who reported high neighborhood violence and disorder compared to those reporting no neighborhood disorder (Crude OR = 1.71, 95%CI = 1.10–2.64). The category of low severity of neighborhood disorder had no significant association with anxiety for Palestinian-Arab women. Adjustment for individual level and neighborhood-level variables and social support in Models 2–4 showed that the association between neighborhood violence and disorder and anxiety did not change much. The final model (Model 5), which was adjusted for chronic stress in addition to the variables in Model 4, rendered the association between neighborhood violence and disorder and anxiety non-significant. High social-cohesion neighborhoods and social support were protective factors from anxiety in Palestinian-Arab women, while aggregate discrimination was a risk factor for anxiety (AOR = 2.39, 95%CI = 1.59–3.60) even after adjustment for chronic stress. Palestinian-Arab women who reported any chronic stress and missing values for chronic stress had higher anxiety compared to women who reported no chronic stress (AOR = 2.58, 95%CI = 2.03–4.82 and AOR = 3.12 (95%CI = 2.03–4.82, respectively) (Table 6).

**Table 6:** Generalized Estimating Equation (GEE) results for the association between neighborhood disorder and violence, and anxiety among Palestinain-Arab women (*N* = 422)

	Model 1	Model 2	Model 3	Model 4	Model 5
	95%CI	95%CI	95%CI	OR (95%CI)	OR (95%CI)
**Neighborhood disorder**					
High severity	1.71 (1.10–2.64)^**^	1.63 (1.08–2.47)^*^	1.58 (1.07–2.35)**	1.52 (1.00–2.30)^*^	1.47 (0.89–2.42)
Low severity	1.12 (0.65–1.92)	1.12 (0.64–1.95)	1.14 (0.66–1.98)	1.13 (0.66–1.93)	1.04 (0.54–1.98)
No problem	1.00	1.00	1.00	1.00	1.00
**Neighborhood SES**					
Low			0.25 (0.01–8.32)	0.24 (0.01–8.14)	0.24 (0.01–4.13)
Medium			0.27 (0.01–9.41)	0.25 (0.01–9.19)	0.24 (0.01–4.43)
High			1.00	1.00	1.00
**Neighborhood aggregate discrimination**					
High			1.77 (1.08–9.92)**	1.80 (1.06–3.05)^**^	**2.39 (1.59**–3.60)^***^
Low			**1.00**	**1.00**	**1.00**
**Social cohesion**					
High			0.48 (0.31–0.74)***	**0.48 (0.31**–0.75)^***^	**0.50 (0.31**–0.81)^**^
Low			**1.00**	**1.00**	**1.00**
**Social group membership**					
At least one group membership			0.91 (0.58–1.41)	0.90 (0.58–1.40)	1.04 (0.62–1.74)
No membership			1.00	1.00	1.00
**Social and political participation**					
Participated at least once			1.05 (0.53–2.07)	1.07 (0.53–2.16)	1.02 (0.51–2.07)
Never participated			1.00	1.00	1.00
**Social support**					
High				0.45 (0.30–0.64)^***^	**0.45 (0.31**–0.65)^***^
Low				**1.00**	**1.00**
**Chronic Stress**					
Missing					**2.58 (2.03**–4.82)^***^
High					3.12 (2.03–4.82)^***^
Low					**1.00**
Model fit	552.41	548.55	542.57	542.57	514.76

***P ≤ 0.001; **P ≤ 0.010; *P ≤ 0.050 (bold = statistically significant associations). Model 1 is unadjusted;

Model 2 controls for individual-level attributes (previous depression and unemployment);

Model 3 controls for individual-level attributes and neighbohood-level characteristics; and

Model 4 adjusts for social support in addition to variables in Model 3.Model 5 adjusts for chronic stress in addition to variables in Model 4

## Discussion

This study is among few to examine residential segregation health effects by exploring associations between neighborhood violence and disorder and ethno-national inequalities in anxiety among perinatal women. We know of no studies that compared anxiety between women in Israel’s two largest ethno-national groups. Also, little research has examined the association between neighborhood violence and disorder and mental health in women in general and during perinatal period [33, 34, 51]. Most studies on anxiety in women looked at individual-level risk factors only [6, 23, 55]. We found that high compared to no neighborhood violence and disorder was associated with almost 1.5 fold anxiety in our total sample of perinatal women after accounting for individual-level factors. High-SES neighborhoods, as well as high social-cohesion and social support were protective factors from anxiety, while aggregate discrimination in a neighborhood was a risk factor for anxiety. When chronic stress was considered, the association between neighborhood violence and disorder and anxiety was reduced and became non-significant for high but not low severity of neighborhood violence and disorder. These results for the total sample support our study hypotheses, showing the contribution of residential segregation, as measured by neighborhood disadvantage (low SES, low social cohesion and high aggregate discrimination), to neighborhood violence and disorder, via chronic stress and lack of social support and, in turn, to anxiety.

A main finding of this study was the large health and residential inequality between the two ethno-national groups of women, a finding that supports our first hypothesis. Palestinian-Arab women reported higher neighborhood violence and disorder, and the prevalence of anxiety was almost 1.5 folds as high among them compared to Jewish women, even after adjustments for individual-level and neighborhood-level characteristics (OR = 1.63, 95%CI = 1.04–2.56).

Looking at the association between neighborhood violence and disorder and anxiety, in each ethno-national group we found that among Jewish women, only the category of low severity of neighborhood violence and disorder was associated with higher anxiety, while among Palestinian-Arab women, the category of high severity of neighborhood violence and disorder was associated with higher anxiety and this association was rendered insignificant following adjustment for chronic stress. This might be explained by the fact that we found more Palestinian-Arab women who reported higher severity of neighborhood violence and disorder, while for Jewish women, low severity of neighborhood violence and disorder was more often reported than high severity. Also, it might be that Jewish women are affected by even low levels of violence and disorder in their neighborhoods, while Palestinian-Arab women might not consider low severity of neighborhood violence and disorder remarkable, as the level of violence and disorder in their neighborhoods is very high in recent years [31]. Palestinian-Arab community has witnessed never-before-seen crime and community violence. A study conducted among Palestinian-Arab adolescents in Israel showed that about two thirds had experienced community violence [31].

Previous research suggested that the ethno-national divide might have led to residential segregation, and was associated with health inequalities between Jewish and Palestinian-Arab citizens [15, 18]. In the current study, residential segregation measured by neighborhood low SES, aggregate discrimination and low social cohesion was associated with higher anxiety among Palestinian-Arab women compared to Jewish women via increased chronic stress and low social support.

In our sample, residential segregation might be a major source of chronic stress in Palestinian-Arab women. The Palestinian-Arab community in Israel has undergone rapid urbanization [36]. However, urbanization was not supported by governmental urban planning [66], leading to conflicts and ‘urbanized villages,’ as many Palestinian-Arabs remain in their home villages due to restrictions on internal migration. Jewish neighborhoods are not very open to including Palestinian-Arab residents, and many Palestinian-Arabs have reported being discriminated against when trying to rent a house or apartment in a Jewish town [53], which was a source of anxiety and depression in Palestinian-Arab men [17]. Now, the current study results show that this might be a source of anxiety in women in this community as well.

In addition, higher anxiety in Palestinian-Arab women compared to Jewish women, might have other explanations. First, prevalence of anxiety was very high in all women in our sample compared to findings from a systematic review in other countries, where anxiety ranged from 5 to 19%, and about 15% in the first year postpartum [22]. Excess of anxiety among women in Israel might be related to different measurement tools of anxiety, but also to long-lasting political violence [9] in addition to issues accompanying pregnancy and birth that are faced by perinatal women everywhere [22, 56]. Our data collection took place during the Gaza-Israel political conflict in 2014–2015 [67]. While this might have affected levels of stress in all women in Israel, as was shown by a previous study on PTSD [9], it might have more greatly affected Palestinian-Arab women, since PTSD was higher at that time among Palestinian-Arab citizens (25%) compared to Jewish Israelis (10%) [9]. Also, while we found that chronic stress was a risk factor for anxiety in both groups, chronic stress was more strongly associated with anxiety in Palestinian-Arab women (AOR = 3.12) compared to Jewish women (AOR = 2.06).

Previous research showed that chronic stress is a risk factor for anxiety among low-income women [33]. Study results from Whitley & Prince [72] who analyzed data from low-income women in the UK support our results regarding the impact of chronic stress. They found that neighborhood disorder might lead to more fear of walking in the streets at certain times, for example at night, and this fear might elevate stress, which might increase anxiety [72]. Also, low-income women living in neighborhoods characterized by high violence had more limitations in using neighborhood services and facilities due to fear, which might be another source of stress [72]. Our finding that chronic stress was associated with attenuation in the association between neighborhood violence and disorder and anxiety in the final models. Future research based on a longitudinal study framework could reveal whether chronic stress is indeed a mediator for the association between neighborhood disorder and anxiety.

The protective effect of social support was notable in the multivariable analysis but it partially confirms our hypothesis. Social support was a protective factor from anxiety in each ethno-national group of women and it reduced the inequalities in anxiety between Palestinian-Arab and Jewish women in the study. However, social support had only little effect on the association between neighborhood violence and disorder and anxiety. This might be explained by the fact that our measure of social support is individual and it had small buffering effect on the neighborhood level of violence and disorder.

The protective effect of social cohesion from anxiety also partially confirms our second hypothesis regarding the role of the neighborhood social attributes. Social cohesion was the only component of the collective efficacy measures that was significant in the multivariable analysis. Neither informal social control, nor the two measures of social capital (social-group membership and social and political participation) were significant in the final multivariable models of anxiety and neighborhood disorder. Despite this increased social cohesion was associated with reduced anxiety in all women in our sample.

We found that low and medium -SES neighborhoods had a protective effect from anxiety for the total sample and low SES neighborhoods had protective effect from anxiety for Jewish women, but not for Palestinian-Arab women. While this result confronts our hypothesis regarding the contribution of higher neighborhood SES to lower anxiety, we think that this result might indicate little overlap in neighborhood SES scales between the two ethno-national groups of women in our study. Similar results were observed in previous studies in the US, where SES in different groups was far apart and could not be compared [2]. We found little overlap between Palestinian-Arab women and Jewish women neighborhood SES, as most Jewish women in the study lived in medium-to-high SES neighborhoods. Also, since all Palestinian-Arab women live in economically disadvantaged areas characterized by low SES, there were no differences in anxiety by neighborhood SES in the final model for Palestinian-Arab women. This suggests that improving the SES of Palestinian-Arab neighborhoods might decrease anxiety associated with neighborhood violence and disorder and, by that, reduce ethnic inequalities in anxiety [18].

Neighborhood aggregate discrimination as a risk factor for anxiety was another important result that was significant only for Palestinian-Arab minority women, not Jewish women. Aggregate discrimination in a neighborhood was associated with almost 2.4-fold more anxiety in Palestinian-Arab women in the final fully adjusted model adjusted. This confirms, in part, our second hypothesis, with Palestinian-Arab women reporting higher ethno-national discrimination compared to Jewish women [19]. In the current study, high neighborhood aggregate discrimination was around 70% among Palestinian-Arab women, 35% in Jewish women.

### Study limitations

Despite our robust study design and study measures, we should mention some limitations. First, our study’s cross-sectional design does not permit causal inference of the tested associations. Another familiar limitation in neighborhood studies and mental health relates to same-source bias. That is, women who have higher poor mental health might report higher neighborhood violence and disorder. While we cannot determine whether reports of high neighborhood disorder and anxiety among women in our sample were related to previous depression, adjusting for taking antidepressants in the multivariable models might have addressed this. Another limitation relates to Palestinian-Arab women’s higher unemployment, which means they are more often home to witness and report violence and disorder than their more-often employed Jewish counterparts. As well, most of our neighborhood characteristics measures were subjective, although we used an objective, Census data based SES measure. While a systematic review of studies found that both subjective and objective measures of neighborhood characteristics were associated with mental health [68], future studies in this area might consider using more objective measures of the neighborhood environment, such as police reports on crime and violence, etc. [59].

## Conclusion

Living in highly segregated neighborhoods characterized by high violence and disorder was associated with higher anxiety and with higher among Palestinian-Arab minority women, who live in ethno-nationally divided neighborhoods, towns and villages from Jewish majority women. This was the case above and beyond individual level-factors. Increased neighborhood disorder thus plays an important role in explaining inequalities in anxiety between these groups. Neighborhood social cohesion and social support were protective factors from anxiety for all women. Yet, living in low-SES neighborhoods was a protective factor from anxiety for Jewish women only, while aggregate discrimination was a risk factor for anxiety among Palestinian-Arab women only. Reversing discriminatory government policies towards Palestinian-Arab neighborhoods might improve neighborhood SES, neighborhood social cohesion and social support, while reducing aggregate discrimination and decreasing violence and disorder in these neighborhoods. This could reduce chronic stress and, in turn, anxiety and eventually inequalities in anxiety between the two ethno-national groups of women.

## Data Availability

All data generated or analyzed during this study are included in this published article [and its supplementary information files].

## Abbreviation

SES: Socioeconomic status
